# Mitigating Ammonia and Greenhouse Gaseous Emission From Arable Land by Co-application of Zeolite and Biochar

**DOI:** 10.3389/fpls.2022.950944

**Published:** 2022-06-30

**Authors:** Awais Ali, Muhammad Fraz Ali, Talha Javed, Syed Hussain Abidi, Quratulain Syed, Usman Zulfiqar, Saqer S. Alotaibi, Dorota Siuta, Robert Adamski, Paweł Wolny

**Affiliations:** ^1^Department of Agronomy, Faculty of Crop and Food Sciences, PMAS Arid Agriculture University, Rawalpindi, Pakistan; ^2^Pakistan Council of Scientific and Industrial Research Laboratories Complex, Lahore, Pakistan; ^3^Department of Agronomy, University of Agriculture, Faisalabad, Pakistan; ^4^College of Agriculture, Fujian Agriculture and Forestry University, Fuzhou, China; ^5^Pakistan Council of Scientific and Industrial Research Laboratories Complex, Islamabad, Pakistan; ^6^Department of Biotechnology, College of Science, Taif University, Taif, Saudi Arabia; ^7^Faculty of Process and Environmental Engineering, Łódź University of Technology, Łódź, Poland

**Keywords:** abiotic stress, biochar, zeolite, ammonia, greenhouse gaseous emission

## Abstract

The intensive use of chemical fertilizers in arable farming dramatically increased environmental pollution through anthropogenic ammonia (NH_3_) and greenhouse gaseous emissions. Therefore, there is a need to develop improved fertilizer management practices that can reduce these losses. An experiment was conducted to assess the mitigating effects of sole or combined application of zeolite with biochar on gaseous emissions from arable land. For this purpose, zeolite (clinoptilolite) was mixed with different doses of biochar (produced from *Dalbergia Sissoo* wood chips) and applied along with the recommended dose of chemical fertilizer (NPK @ 150, 100, and 60 kg ha^–1^, respectively) on arable land in years 2013–14 and 2014–15. Immediately after application, these were incorporated into the top 10 cm of the soil layer and wheat was sown. Treatments were as follows: C = control, Z = zeolite @ 5 t ha^–1^, B1Z = biochar @ 3 t ha^–1^ + zeolite @ 5 t ha^–1^, B2Z = biochar @ 6 t ha^–1^ + zeolite @ 5 t ha^–1^, and B3Z = biochar @ 9 t ha^–1^ + zeolite @ 5 t ha^–1^. The experiment was laid out in a randomized complete block design (RCBD) with three replicates. The experimental plot size was 6 m × 4 m. Randomly, ten soil samples from each plot were taken at a depth of 0–15 cm and mixed to get a composite sample. All the samples were immediately stored in a freezer at −18°C until gaseous analysis in order to prevent N transformations. Each soil sample was analyzed for emission of NH_3_, CO_2_, and CH_4_ by using a selected-ion flow-tube mass spectrometer (SIFT-MS). Co-application of zeolite and biochar reduced NH_3_ and CH_4_ emissions by an average of 87 and 58% compared to the control, respectively. However, CO_2_ emission was increased by 104% relative to the control. The NH_3_ emission was decreased by an average of 61, 78, 90, and 92% by Z, B1Z, B2Z, and B3Z treatments compared to the control. Similarly, the decrement in CH_4_ emission was 47, 54, 55, and 65%. In contrast, the increment in CO_2_ emission was 42, 110, and 160% for B1Z, B2Z, and B3Z, respectively, while interestingly, a reduction of 12% was observed in Z treatment. Besides, co-application of zeolite and biochar at the highest dose (B3Z) improved soil chemical properties such as soil EC, OM, total N, as well as available P and K relative to zeolite alone. It is concluded that the combined application of zeolite and biochar can mitigate NH_3_ and greenhouse emissions and improve soil chemical characteristics, thus enhancing the environmental worth of arable farming.

## Introduction

The application of chemical fertilizers to arable land in Pakistan has reduced soil carbon (C) inputs, depleted soil nutrients, increased soil erosion, ammonia (NH_3_) and greenhouse gas (GHG) emissions, and decreased soil microfauna (Mohammad et al., 2012; Lal, 2018). In Punjab province alone, 1.58 million hectares of soil are strongly nutrient deficient (Raheem et al., 2010). Application of urea and other chemical fertilizers to arable land causes anthropogenic emissions of GHG, including nitrous oxide (N_2_O), carbon dioxide (CO_2_), and methane (CH_4_), which contribute to polluting our global environment (Benbi, 2013; Fahad et al., 2015a; Al-Zahrani et al., 2022). Besides, nitrogen (N) loss in the form of NH_3_ might enhance acidification and eutrophication of terrestrial and aquatic ecosystems after dry and wet deposition (Yan et al., 2003; Khan and Mohammad, 2014). Hence, low-cost, ecologically friendly, and socially acceptable techniques for fertilizer management are necessary for modern agriculture (Fahad et al., 2016a).

The use of biochar and zeolite due to their porous structure and absorbent nature can be considered a promising practice, especially because of their potential to retain NH_3_ through ammonium (NH_4_^+^) adsorption (Fahad et al., 2015d,^2018^, ^2020^, 2021a). Biochar is a carbonaceous material obtained through pyrolysis of plant biomass in the absence of oxygen (López-Cano et al., 2018; Bamagoos et al., 2021). Additionally, biochar also leads to C sequestration of approximately 50% of the initial C, therefore when applied in soil yielding more stable C. Biochar when applied in soil reduced N_2_O emissions by up to 90% while CH_4_ emissions by up to 52% (Spokas et al., 2009; Woolf et al., 2010; Fahad et al., 2015c; Adekiya et al., 2019). However, studies on the emission of CO_2_ from the soil with the application of biochar along with other fertilizers showed contrasting from positive to negative or even no effects depending upon the texture of the soil (Novak et al., 2010; Scheer et al., 2011; Wang et al., 2014). The addition of biochar stimulates the mineralization of soil organic carbon (SOC) and ultimately increases emissions of CO_2_ (Luo et al., 2011; Fahad and Bano, 2012; Hussain et al., 2017). Furthermore, the application of biochar facilitates microbial growth with higher soil respiration as compared to unamended soil, which results in enhanced CO_2_ emission (Fahad et al., 2016c; Shah et al., 2017; Xiang et al., 2017). Biochar produced from different feedstocks did not always result in a win-win scenario, since many studies ended up with conflicting outcomes for its potential to mitigate GHG emissions and improve crop yield (Woolf et al., 2010; Agegnehu et al., 2016; Haider et al., 2017). This means that biochar might not always be fruitful for mitigating GHG emissions when applied to cultivated soil.

Recently, zeolite is considered a good tool for mobilization of plant nutrients and mitigation of NH_3_ and GHG emissions from arable land (Aziz et al., 2019). Zeolite is a Greek word that means boiling stone; it was first discovered in 1,756 by Swedish mineralogist Baron Alex Frederick. It has crystalline-hydrated characteristics resulting from its infinite three-dimensional structures, which make it a strong adsorbent (Ndegwa et al., 2008; Fahad et al., 2015a,b). The use of zeolite is increasing day by day in agriculture, medicine, industry, and environmental protection (Van Straaten, 2006). Clinoptilolite (Na, K)_6_[Al_6_Si_30_O_72_] 20H_2_O) is one of the well-known zeolites widely used in agriculture due to its hydration and dehydration ability without changing its structure, catalysis, and cation exchange capacity. The N losses from the soil in the form of NH_4_^+^ and nitrate (NO_3_^–^) through leaching are common phenomena in poor soils due to their low cation exchange capacities. Besides, NH_3_ volatilization from poor soils has been estimated to be 1–60% of the total applied N (Haruna Ahmed et al., 2008; Fahad et al., 2021e). Wang et al. (2012) found that the application of zeolite to stored duck manure significantly reduced NH_3_ and CH_4_ emissions. The use of zeolite can significantly reduce NH_3_ emission from the soil, which ultimately increases N use efficiency and hence crop yield (Fahad et al., 2013; Aziz et al., 2019; Fahad et al., 2021d). The use of zeolite along with chemical fertilizers might mitigate NH_3_ and GHG gaseous emissions, but it might result in a serious decline in soil organic matter, hence its fertility due to their non-organic C nature. Thus, the integrated application of zeolite with biochar could be a suitable strategy for mitigating NH_3_ and GHG emissions, improving crop yield as well as C sequestration and microbial biomass in the soil. Therefore, the objective of the study was to assess the mitigating effects of the sole or combined application of zeolite with biochar on gaseous emissions from arable land. Besides, their effects on soil chemical characteristics were also evaluated.

## Materials and Methods

This study was performed at the university research farm, Chakwal Road, Koont of PMAS Arid Agriculture University, Rawalpindi, Pakistan, in the years 2013–14 and 2014–15. This farm is located in Chakwal (latitude of 32.9303°N, longitude of 72.8558°E, and altitude of 2,500 feet), Pakistan. The climate of the region is local steppe according to the classification of Koppen (1936). In summer, the temperature of the region reaches 40°C; however, in winter, it remains between −3 and 25°C, while rainfall varies between ∼7 and 137 mm during the year (PMD, 2012). The air temperature in the Rabi season during this experiment was between 8 and 32°C in the years 2013–14 and 2014–15, with rainfall ranging from 35 to 254 mm. The texture of the soil was sandy clay loam. According to the classification of the Government of Pakistan, the soil belonged to the Rawal series and was categorized as Udic Haplustalf Alfisols (Digar et al., 1973). The experimental plot size was 24 m^2^ (6 m × 4 m), and the plot-to-plot distance was 1 m, while a distance of 2 m was kept between replications. A randomized complete block design (RCBD) with 5 treatments and three replications was used. Treated soil was cultivated for 2 years under wheat crop and then the soil was tested for the effect of co-application of zeolite and biochar on NH_3_ and GHG emissions from arable land where chemical fertilizers were applied. Natural zeolite (clinoptilolite) in powder form was purchased from Mehran Mining Company (Pvt. Limited), Karachi, Pakistan, and was distributed by Hassan Enterprises located in Province Khyber Pakhtunkhwa, Pakistan (Aziz et al., 2019).

The soil was plowed before the onset of the monsoon season to conserve moisture with a chisel plow at the start of June 2013 and 2014. The seedbed was prepared by tine plow (3 times) with a planker to improve soil tilth. Treatments were as follows: C = control, Z = zeolite @ 5 t ha^–1^, B1Z = biochar @ 3 t ha^–1^ + zeolite @ 5 t ha^–1^, B2Z = biochar @ 6 t ha^–1^ + zeolite @ 5 t ha^–1^, and B3Z = biochar @ 9 t ha^–1^ + zeolite @ 5 t ha^–1^. Immediately after the incorporation of zeolite and biochar into the top 10 cm of the soil layer, wheat (*Triticum aestivum*) variety Chakwal-50 was sown at the rate of 130 kg ha^–1^ by using a manual seed drill. Recommended rates of NPK fertilizers (N: 150, P_2_O_5_:100, and K_2_O:60 kg ha^–1^) were used in each treatment (Ali et al., 2015). All other cultural practices were kept similar in all plots to evaluate the effect of treatments on soil chemical characteristics and gaseous emissions.

### Production of Biochar

Biochar was produced by using a methane gas-driven biochar production unit (conventional pyrolysis tank) in the Institute of Soil Science at PMAS-Arid Agriculture University, Rawalpindi, Pakistan, by using *Dalbergia sissoo* (Rosewood) wood chips collected from a local furniture factory as described by Sadaf et al. (2017). Wood chips were pyrolyzed at 350–400°C (slow pyrolysis), resulting in a 45–50% biochar yield. After preparation, biochar was ground to pass a 2 mm sieve prior to its field application.

### Chemical and Structural Analysis of Zeolite and Biochar

**FIGURE 1:**
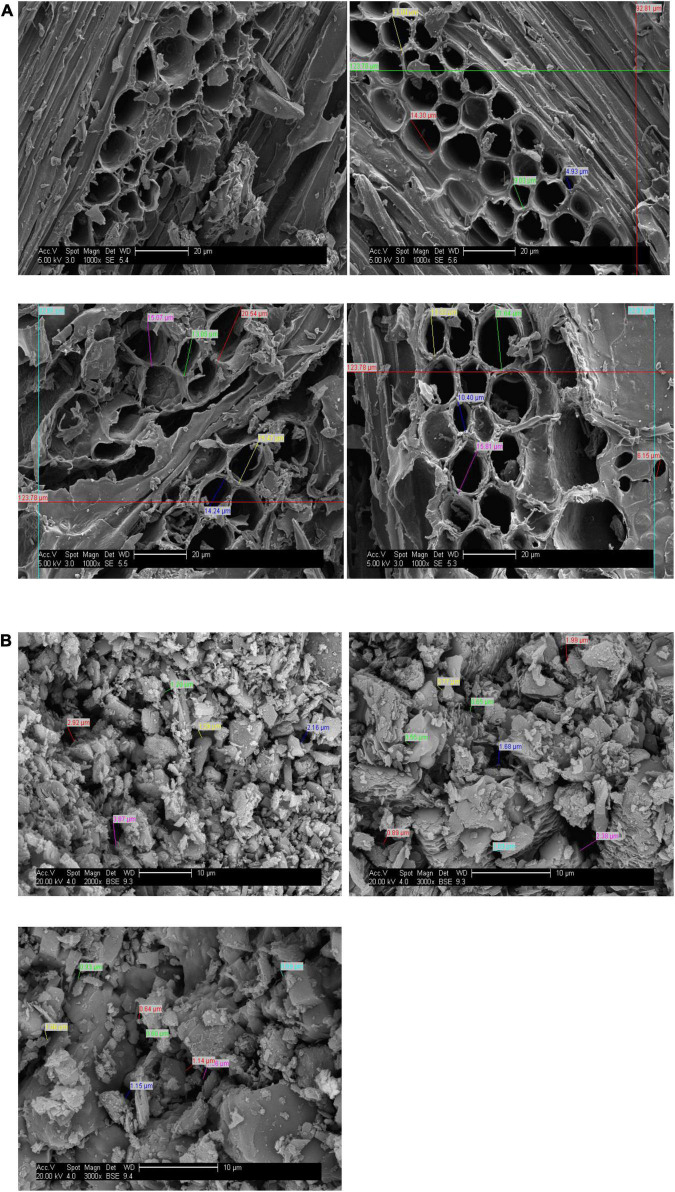
Scanning electron microscope (SEM) photographs of panel **(A)** biochar produced from *Dalbergia sissoo* and **(B)** zeolite (clinoptilolite) showing their structure.

The zeolite and biochar used in this experiment were analyzed for their chemical properties in a laboratory by adopting standard operating procedures. The results are shown in Tables 1, 2. Structural analysis was done by using a scanning electron microscope (Philips XL-30SFEG), and the results are presented in Figures 1A,B.

**TABLE 1:** Mean (*n* = 3) of the physiochemical characteristics such as bulk density (BD), pH, electrical conductivity (EC), carbon (C), oxygen (O), phosphorous (P), potassium (K), silicon (Si), and C:O ratio of biochar produced from *Dalbergia sissoo*.

Parameter	Unit	Value
BD	g cm^–3^	0.31
pH	–	8.50
EC	dS m^–1^	1.21
C	%	63.16
O	%	25.03
P	%	0.27
K	%	0.65
Si	%	6.18
C:O	–	2.52

**TABLE 2:** Mean (*n* = 3) of the physiochemical characteristics such as bulk density (BD), pH, cation exchange capacity (CEC), silicon (Si), aluminum (Al), magnesium (Mg), calcium (Ca), iron (Fe), phosphorous (P_2_O), and potassium (K_2_O) of zeolite (clinoptilolite) used in the experiment.

Parameter	Unit	Value
Bulk density	g cm^–3^	0.79
pH	–	7.7
CEC	cmol kg^–1^	92.1
Si	%	33.9
Al	%	5.09
Mg	%	0.28
Ca	%	0.32
Fe	%	1.22
P_2_O	mg kg^–1^	2.12
K_2_O	mg kg^–1^	3.13

### Measurement of Ammonia and Greenhouse Gas Emissions

**FIGURE 2:**
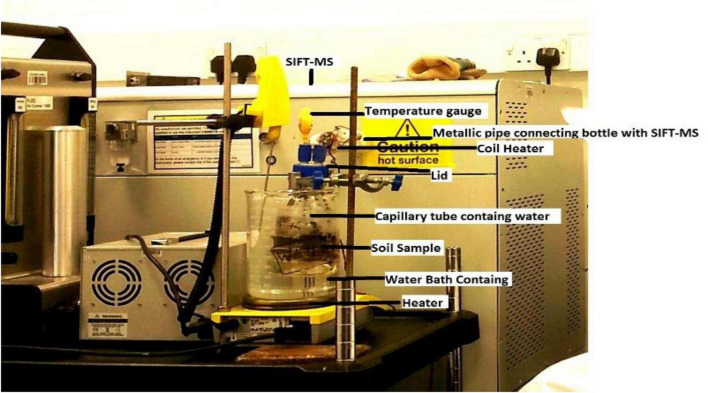
Photograph of the selected-ion flow-tube mass spectrometer (SIFT-MS) showing the analysis procedure for the measurement of gaseous emissions from soil samples taken from the arable land.

Each year, at the end of the experiment, ten soil samples randomly from each plot were taken at a depth of 0–15 cm and mixed to get a composite sample. All the samples were immediately stored in a freezer at −18°C until gaseous analysis in order to prevent N transformations (Shah et al., 2016). The samples were transported with extreme care to Canfield University, United Kingdom, and analyzed for gaseous emissions. Each soil sample was thawed at room temperature and analyzed for emission of NH_3_, CO_2_, and CH_4_ by using a selected-ion flow-tube mass spectrometer (SIFT-MS; Figure 2).

The technique was used to measure volatile compounds and trace gases. It was based on ionization by using H_3_O^+^, O_2_^+^, and NO^+^ precursor ions *via* ionic or molecule reactions taking place inside SIFT-MS for a specific period of time. The concentration of volatile compounds was calculated in mg kg^–1^ of soil (Milligan et al., 2002). A 100-g thawed soil sample was put into a glass bottle and closed with a lid. The bottle was connected to SIFT-MS by a metallic pipe from one side and opened into the bottle from the other side. A small capillary tube filled with 20 ml of demineralized water was placed inside the bottle to maintain humidity. A bottle was dipped into a hot water bath with a constant temperature of 30°C. When soil inside the bottle becomes hot, it releases volatile compounds and trace gases, which were detected by SIFT-MS. Volatile compounds emitting out of the soil were shown on a digital screen as compared to a free air sample. The volatilization of NH_3_, CO_2_, and CH_4_ was determined from each soil sample (Figure 2).

### Chemical Analysis of Soil Samples

Each composite soil sample was analyzed for pH, electrical conductivity (EC), total nitrogen (N), available phosphorous (P), extractable potassium (K), and organic matter (OM). Soil pH and EC were measured from the soil and water suspension (1:2.5) that was equilibrated for 30 min at room temperature. After that, a multimeter (Ino-Lab^®^ Multi 9430 IDS, WTW, GmbH & Co., KG, Germany) was standardized with 0.01 N KCl at 25°C, and values of these parameters were recorded from each treatment. Soil total N content was determined by the Kjeldahl digestion method. Soil available P and K were determined according to the procedures described by Houba et al. (1986). The carbon content of the soil samples was measured through wet oxidation of the samples by means of chromic acid, hydrogen peroxide, and sulfuric acid (Walkley and Black, 1934), as carried out by Agegnehu et al. (2016) for carbon determination in soil.

### Statistical Analysis

For this experiment, we used a two-way linear model analysis of variance. Data collected from this experiment were analyzed statistically by using Statistix (version 8.1). A least significant difference (LSD) test with a 5% probability level was applied to compare the treatments’ mean (Steel et al., 1997).

## Results

**FIGURE 3:**
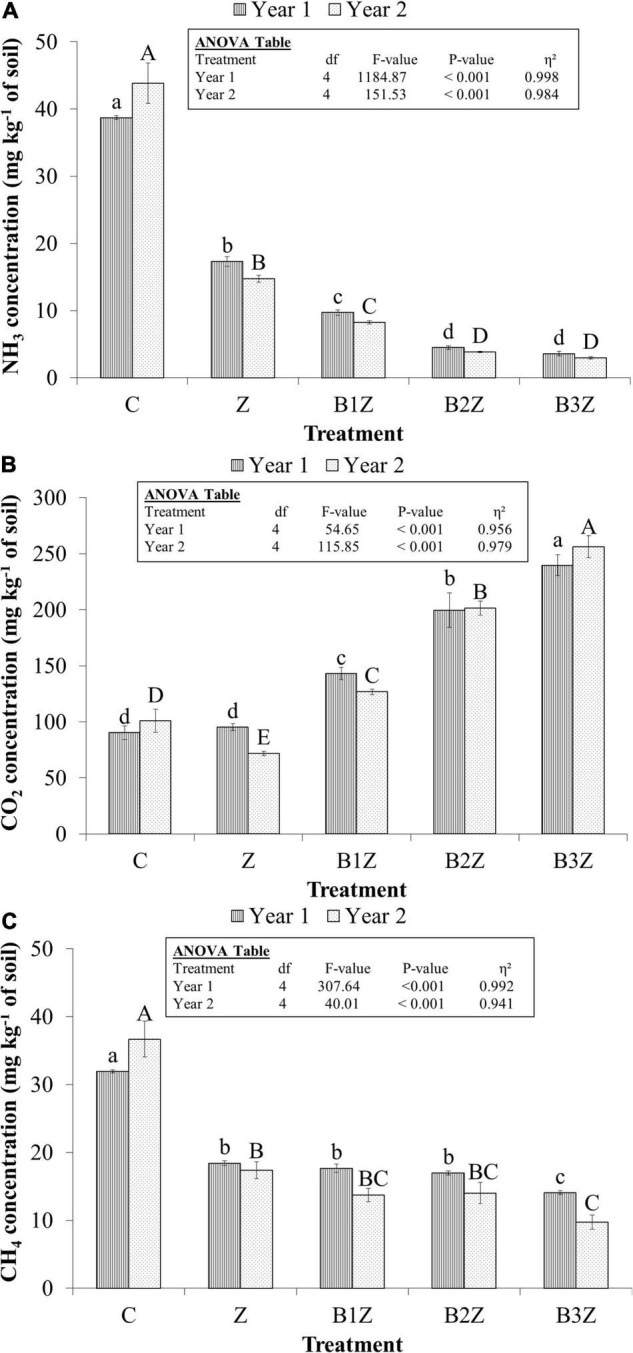
Emissions of panel **(A)** NH_3_, **(B)** CO_2_, and **(C)** CH_4_ from arable land after co-application of zeolite and biochar along with chemical fertilizers. Error bars represent the standard error (±) of the mean. Data bars of each parameter having dissimilar small or capital letters differed significantly from each other (*P* ≤ 0.05). The inset table indicates the analysis of variance (ANOVA). Multiple comparisons among treatments were analyzed by LSD test. C = control, Z = zeolite @ 5 t ha^– 1^, B1Z = biochar @ 3 t ha^– 1^ + zeolite @ 5 t ha^– 1^, B2Z = biochar @ 6 t ha^– 1^ + zeolite @ 5 t ha^– 1^, B3Z = biochar @ 9 t ha^– 1^ + zeolite @ 5 t ha^– 1^.

Overall, emission of NH_3_ and CH_4_ was significantly reduced by 87 and 58%, while CO_2_ was increased by 104% after co-application of zeolite and biochar relative to control under an inorganic farming system, respectively (*P* ≤ 0.05; Figures 3A–C). Sole application of zeolite mitigated NH_3_, CO_2_, and CH_4_ emissions on average by 61, 47, and 12% compared to the control. However, better results were found with the combined application of zeolite and biochar for the decrement in NH_3_ and CO_2_. In contrast, the combined application enhanced CO_2_ emissions. The process accredited this to NH_4_^+^ adsorption on the cation exchange surface of zeolite having a high cation exchange capacity of 92.1 cmol kg^–1^ (Table 2).

The reduction in NH_3_ emission was 55 and 85% by the sole and combined application of zeolite with biochar in year 1, while the aforementioned reduction was 66 and 89% in year 2, respectively (Table 3). Similar trends were observed in CH_4_ emission, with a decrement of 42 and 53% in year 1 and 49 and 66% in year 2, respectively. Contrarily, CO_2_ emission was enhanced by 5% in year 1 while reduced by 29% in year 2 after the sole application of zeolite. However, the combined application of zeolite with biochar increased its emission by 115 and 93% in years 1 and 2, respectively (Table 3).

**TABLE 3:** Relative gaseous losses compared to control from arable land after co-application of zeolite and biochar along with chemical fertilizers.

Treatments	NH_3_		CO_2_		CH_4_	
	Year 1	Year 2	Year 1	Year 2	Year 1	Year 2
C	100	100	100	100	100	100
Z	45	34	105	71	58	47
B1Z	25	19	158	126	55	37
B2Z	12	09	221	200	53	38
B3Z	09	07	265	254	44	27

*C = control, Z = zeolite @ 5 t ha^–1^, B1Z = biochar @ 3 t ha^–1^ + zeolite @ 5 t ha^–1^, B2Z = biochar @ 6 t ha^–1^ + zeolite @ 5 t ha^–1^, B3Z = biochar @ 9 t ha^–1^ + zeolite @ 5 t ha^–1^.*

The highest reduction in NH_3_ and CO_2_ emissions by 92 and 44% was observed in B3Z treatments compared to all others during both years of study (Table 3). The NH_3_ emissions through zeolite and biochar amended treatments were 9, 4.2, and 3.3 mg kg^–1^ of soil for B1Z, B2Z, and B3Z, respectively (Figure 3A). The emissions of CH_4_ from the aforementioned treatments were 15.7, 15.5, and 11.9 mg kg^–1^ of soil, respectively (Figure 3C). The respective values for CO_2_ were 135, 201, and 248 mg kg^–1^ of soil (Figure 3B). Application of only zeolite resulted in NH_3_, CH_4_, and CO_2_ emissions by 16, 18, and 84 mg kg^–1^ of soil, respectively. The CO_2_ emission was enhanced with the application of biochar, and emission fluxes seem to have a direct relationship with the applied dose of biochar. The highest CO_2_ emission was observed at biochar @ 9 t ha^–1^ + zeolite @ 5 t ha^–1^, with a 165% increment over control in year 1 and 154% in year 2 (Table 3).

Soil pH is one of the key factors, which stimulate the availability of nutrients to crops. Soil with an extremely low or high pH badly affects plant growth. In our experiment, a minimum soil pH (7.36) was observed in the treatment biochar B1Z. Zeolite treatment has a slight increase of 0.2% in soil pH in both experimental years. Interaction of biochar and zeolite significantly enhanced soil pH. It was observed that treatments B2Z and B3Z showed a maximum increase of 0.4% in soil pH during both experimental years (Table 4). Results calculated from both years revealed that biochar can change soil pH, whereas zeolite treatments have no effect as compared to biochar treatments (Table 4).

**TABLE 4:** Mean of 2 years (*n* = 3) soil pH, electrical conductivity (EC), total nitrogen (N), available phosphorous (P), extractable potassium (K), and organic matter (OM) of soil as influenced by the application of zeolite (Z) alone or in combination with biochar (B) applied at various doses.

Treatments	pH	EC	Total N	Available P	Extractable K	OM
				
		(dS m^–1^)	(mg kg^–1^ of soil)			(%)
Control	7.37	48.4	1.30	2.79	52.48	0.49
Z	7.38	61.6	3.09	7.31	128.04	0.97
B1Z	7.36	58.0	2.87	6.80	107.78	0.87
B2Z	7.40	59.6	2.85	8.35	135.76	1.10
B3Z	7.40	74.7	3.79	9.57	185.81	1.25

*C = control, Z = zeolite @ 5 t ha^–1^, B1Z = biochar @ 3 t ha^–1^ + zeolite @ 5 t ha^–1^, B2Z = biochar @ 6 t ha^–1^ + zeolite @ 5 t ha^–1^, B3Z = biochar @ 9 t ha^–1^ + zeolite @ 5 t ha^–1^.*

Electrical conductivity (EC) is the measure of the concentration of salts and moisture present in the soil. It affects plant nutrient availability, crop suitability, and the activity of soil microorganisms. The application of biochar and zeolite significantly affected the EC of soil as shown in Table 4. Maximum soil EC (74.7) was recorded in treatment B3Z, while minimum (48.4) was recorded in the control (Table 4). Interaction of biochar and zeolite treatments has significantly increased EC by 12%–25%. A maximum increase in EC was observed in treatment B3Z with a 35% increase over control. While sole zeolite application at the rate of 5 tons ha^–1^ has increased the EC by 21.4% over control, which was followed by an increase of 18.79 and 12% by treatments B2Z and B1Z as shown in Table 4.

Nitrogen (N) plays an important role in the vegetative growth of crops as it is one of the most limiting factors in crop growth (Fahad and Bano, 2012; Fahad et al., 2013; Fahad et al., 2015a,b; Fahad et al., 2016a,b,c,d; Fahad et al., 2017). It helps to increase total biomass, and it also affects protein synthesis by the plant. Nitrogen contents were determined at soil depths of 0–15 cm. As shown in Table 4, it was found that application at the rate of by treatment biochar B3Z has a maximum total N (3.79 mg kg^–1^), while a minimum (1.3 mg kg^–1^) was recorded in the control (Table 4). A maximum amount of total N was observed in treatment B3Z, with a 65% increase over control. Sole zeolite treatment Z (5 ton ha^–1^) has increased soil total N from 1.30 to 3.09 mg kg^–1^, which is 57.9% higher than the control. Treatments B1Z and B2Z almost have the same trend for increasing total N by 55.7% and 55.3% as the control. An increase in soil total N might be due to the charged surface, high porosity, and nutrient retention properties of biochar and zeolite.

Phosphorous (P) is one of the essential nutrients required by the plant for optimum growth and reproduction. The deficiency of P can cause serious constraints for the crop, like delayed maturity, reduced biological yield, and decreased disease resistance. Based on the data collected, a significant increase in available P was observed with biochar and zeolite sole and combined application as shown in Table 4. In all the applications, available P was increased from 57 to 72% over control. The maximum (9.57 mg kg^–1^) available P was recorded in B3Z, which was 72% more than the control, and the minimum (2.79 mg kg^–1^) was observed in the control. In the case of sole, zeolite application at the rate of 5 tons ha^–1^ has increased available P by 61.83% over the control. In the combined application of biochar and zeolite, the maximum increase in available P was recorded in B3Z, B2Z, and B1Z with 9.57, 8.35, and 6.80 mg kg^–1^, respectively, as shown in Table 4. Overall, it was observed that combined treatments performed better than sole treatments. Biochar and zeolite, due to their porosity and net negative surface charge, might be responsible for this increase. These negatively charged particles help to retain a variety of nutrients along with moisture. It helps in enhancing nutrient and moisture levels in the soil.

Potassium (K) has vital importance in the plant life cycle. Data regarding extractable potassium are shown in Table 4. A significant difference was observed with the sole and combined application of biochar and zeolite along with control. In sole, zeolite treatment (5 t ha^–1^) showed an average increase of 59% in extractable potassium over control. Results regarding the combined application of biochar and zeolite showed a relative increase of 51.30, 61.34, and 71.64% in B1Z, B2Z, and B3Z, respectively, over control. The increase in K availability might be because biochar and zeolite reduce nutrient leaching and increase fertilizer use efficiency. As K is one of the primary nutrients, which is abundantly required by the plant because it is involved in many physiological processes, e.g., photosynthesis, enzyme activation, and transport assimilation.

Organic matter helps soil in conserving moisture and enhances the availability of nutrients to crops. Data shown in Table 4 reveal that the application of biochar and zeolite has a significant effect on soil organic matter. In sole, zeolite application treatment (5 t ha^–1^) has increased soil organic matter by 52.5% as compared to control. The interaction of biochar and zeolite was also significant. It was observed that all combined treatments somewhat increased soil organic matter. An increase in organic matter is thought to be due to the addition of carbon present in the biochar or an increase in microbial biomass with the addition of biochar and zeolite. The combined application of biochar and zeolite significantly increased soil organic matter. The biochar used in this experiment contains 63% of carbon. An increase in soil carbon ultimately increases organic matter. Therefore, the carbon content of biochar might be responsible for the increase in organic matter content.

## Discussion

The application of biochar and zeolite significantly influenced greenhouse gaseous emission, soil chemical characteristics, and nutrient availability. Our results are in line with Shah et al. (2018) who found that the application of zeolite-amended manure reduced NH_3_ emission by about two-thirds of the manure applied to grassland. Likewise, in an experiment, wheat straw biochar addition in paddy soils reduced N_2_O emission by 42%, but there was a 12% increase in CO_2_ emission (Zhang et al., 2012). Besides, this process may reduce the concentration of NH_3_ (gas) in the soil solution and improve the NH_3_ (water) in it (Ndegwa et al., 2008). High C content in biochar affects their nitrate adsorption capacity, hence mitigating leaching and gaseous N losses (Haider et al., 2017). NH_3_ emission was decreased because biochar and zeolite, due to their structure and charge (net negative charge), can absorb it. Moreover, the reduction in NH3 emission might be due to improved soil aeration and pH, inhibition of nitrification by volatile organic compounds, and adsorption of NH4 + by an increase in CEC (Di et al., 2010; Dempster et al., 2012; Kasper et al., 2019). Biochar regulates greenhouse gaseous emissions in various ways. It can regulate nitrification and denitrification processes by modifying soil properties and microbial activity, thereby inhibiting the production of N2O. First, it enhances soil permeability and inhibits denitrification. Then, it increases soil pH and increases the activity of the N2O reductase enzyme, thus reducing N2O content (Subedi et al., 2016; Fahad et al., 2017). A decrease in CH_4_ emission was due to an increase in the aeration of the soil. Lehmann et al. (2006) reported that the addition of 2% w/w of biochar has the potential to significantly reduce CH_4_ emission compared to untreated control (P ≤ 0.05). They also attributed the reduction in CH_4_ emission to an increase in soil aeration, which discourages the mechanism of methanogenesis (anaerobic condition for CH_4_ production). However, an increment in CO_2_ emission might be due to the decomposition of native soil organic matter (priming effect), labile carbon mineralization, and enhanced aeration, which facilitate respiration of microbial biomass in soil. Besides, microbial activities in soil with biochar addition might be increased, resulting in high CO_2_ emissions (Wardle et al., 2008). Although the use of biochar as a carbon-rich compound increased CO_2_ emission from the soil, this emission was much less compared to natural decomposition in the open atmosphere. The addition of biochar to soil tends to reduce ammonification by reducing NH_3_ volatilization due to its adsorbent nature (Gundale and DeLuca, 2006). It has already been proven that zeolite application can enhance N uptake, crop apparent N recovery and dry matter yield, and mitigate NH_3_ volatilization (He et al., 2002; Shah et al., 2018; Fahad et al., 2021c).

Biochar and zeolite application significantly improved organic matter and nutrient concentration in soil. Cation adsorption and increased pH are the main factors for improved nutrient retention in soil (Jakkula and Wani, 2018; Fahad et al., 2021b). The higher surface charge density of biochar helps to maintain cations for ion exchange, while the higher surface area, porosity, and existence of both polar and non-polar sites help biochar to retain nutrients and organic molecules (Fahad et al., 2016b,d).

Application of biochar might affect soil bulk density because the porosity of biochar is very high, and when it is applied to the soil, it significantly decreases bulk density by increasing pore volume, hence aeration of the soil, creating more aerobic conditions (Fahad et al., 2015d). Besides, the adsorption nature and high porosity of biochar, as well as zeolite, might also increase soil aeration. Consequently, aerobic decomposition of soil organic matter resulted in more CO_2_ and less CH_4_ emissions. An increase in CO_2_ emission might be attributed to the decomposition of native soil organic matter, labile carbon mineralization, as well as enhanced aeration, which facilitates the respiration of microbial biomass in soil. Our results are in line with Van Zwieten et al. (2015) and Xu et al. (2019) who found a reduction in CH_4_ and an increment in CO_2_ emissions after the application of biochar as a soil amendment.

Soil is thought to be a source of biogeneric emission of organic volatile compounds. Microbial and plant (plant roots) activities in soil decomposed organic matter and litter which results in the emission of organic volatile compounds into the atmosphere (Peñuelas et al., 2014). The emission of volatile compounds depends on the presence of volatile compounds in the soil, microbial biomass, and the rate of soil respiration (Mancuso et al., 2015). Production of biochar through biomass was found to be an effective way to dispose of residual biomass and help to reduce greenhouse gas emission (Lehmann and Joseph, 2009; Fahad et al., 2021b; Ul Hassan et al., 2021). N_2_O and CH_4_ are two main components of greenhouse gases, which are associated with the agriculture sector. The reduction in gaseous emissions through amending soil with zeolite and biochar in inorganic farming might improve the environmental value of these systems.

## Conclusion

Co-application of zeolite and biochar in the field remarkably reduced NH_3_ and CH_4_ emissions from arable land; however, CO_2_ emission was enhanced. The results showed that gaseous emissions can be reduced in the long run through the combined application of zeolite and biochar. Likewise, soil chemical characteristics were also improved by these amendments. Moreover, co-application treatments might have positive effects on the nutrient availability to the plants, hence dry matter yield. This study successfully demonstrated that both zeolite and biochar have great potential to be used in arable farming to mitigate NH_3_ and GHG emissions and improve the agro-environmental worth of these systems in the long run. Therefore, it is proposed that the co-application of zeolite, biochar, and chemical fertilizer in intensively managed soils can be the best strategy to achieve long-term benefits in terms of mitigating nutrient losses and improving soil quality in infertile rainfed soils, consequently crop yield.

## Data Availability Statement

The original contributions presented in this study are included in the article/supplementary material, further inquiries can be directed to the corresponding author.

## Author Contributions

All authors listed have made a substantial, direct, and intellectual contribution to the work, and approved it for publication.

## Conflict of Interest

The authors declare that the research was conducted in the absence of any commercial or financial relationships that could be construed as a potential conflict of interest.

## Publisher’s Note

All claims expressed in this article are solely those of the authors and do not necessarily represent those of their affiliated organizations, or those of the publisher, the editors and the reviewers. Any product that may be evaluated in this article, or claim that may be made by its manufacturer, is not guaranteed or endorsed by the publisher.
